# Internet of Things and healthcare system: A systematic review of ethical issues

**DOI:** 10.1002/hsr2.863

**Published:** 2022-10-03

**Authors:** Somayyeh Zakerabasali, Seyed Mohammad Ayyoubzadeh

**Affiliations:** ^1^ Department of Health Information Management, Clinical Education Research Center, Health Human Resources Research Center, School of Health Management and Information Sciences Shiraz University of Medical Sciences Shiraz Iran; ^2^ Department of Health Information Management, School of Allied Medical Sciences Tehran University of Medical Sciences Tehran Iran

**Keywords:** ethical issues, healthcare, Internet of Things, IoT

## Abstract

**Background and Aims:**

The Internet of Things (IoTs) is a set of connected objects and devices that share data and pursue a common goal in different areas. IoT technology can significantly help the healthcare system by enabling the monitoring of elderly and chronic disease patients. Along with the growth of this technology, its challenges and limitations such as Connectivity, Compatibility, Standards, cost, legal, and ethical also increase. One of the most critical and challenging issues in the IoT is ethical issues. This study aims to explore the key ethical aspects of the IoT and Categorize them based on the executive phases of IoT in healthcare.

**Methods:**

The current study was conducted in two phases using the mixed‐method approach. In the first phase, a systematic review was conducted in relevant databases to identify ethical issues of the IoT. In the second phase, a focus group discussion was conducted to classify the extracted data elements based on executive phases of IoT by medical informatics experts and computer engineerings.

**Results:**

Among the 138 papers retrieved through the search strategy, 11 articles were selected, and 12 ethical issues related to IoT were identified. The obtained results revealed the importance of ethical issues of IoT, including security, confidentiality, privacy, anonymity, freedom to withdraw, informed consent, integrity, availability, authorization, access control, censoring, and eavesdropping. They were classified into five main categories of executive phases of IoT based on the five experts’ opinions affiliated with SUMS, including data collection, data storage, data process, data transmission, and data delivery.

**Conclusion:**

Because of the key role of the IoT in disease prevention, real‐time tele‐monitoring of patient's functions, testing of treatments, health management, and health research, considering the risks relating to Health care and patient data is essential. Moreover, health policymakers should be aware of the ethical commitment to using IoT technology.

## 1 INTRODUCTION

The Internet of Things (IoT) provides the concept of the smart world that things will be able to interact with other things by connecting to the Internet or with the help of communication tools and sharing their information with each other or humans. IoT provides new classes of capabilities, applications, and services to help people. It will be a world in which all things and heterogeneous devices will be able to have addresses and controllability.^1^ The IoT will be considered a future innovation in wireless technologies and will be applied in many areas. It is defined from different perspectives.^2^ For instance, from the viewpoint of^3^:

1. Services provided by things: is “the world in which all things can automatically communicate with computers and provide all services for the benefit of humans.”^4^
2. Connection: is “the ability to communicate with anyone or anything at any time or in any place.”^5^
3. Communications: “an extensive network of interconnected objects with unique addresses based on standard communication protocols.”^6^
4. Networks: is “an internet developed from a network of interconnected computers to a network of interconnected objects.”^7^

One of the important applications of the IoT in various medical areas is remote patient monitoring systems and emergency alert systems, and follow‐up of discharged patients. Since monitoring the health parameters of patients is done through sensors on their bodies, IoT may allow patients to stay in various places, such as their homes, workplaces, public places, or vehicles, while medical sensors are still attached to them and transferring information to the medical center.^8^ Health care systems can provide many advantages of IoT, such as the patient with chronic diseases monitoring, monitoring the elderly, and receiving quick medical responses from physicians. As a result of this, hospital costs will be dramatically reduced through immediate intervention and quick treatment.^2^, ^9^ The main goal of IoT in the electronic health system is to help current healthcare monitoring systems through real‐time and online monitoring of vital signs and health data for the patient. In this approach, complete and precise data transfer from patients to medical centers is essential.^10^ Failure to do this might jeopardize the patient's life. One of the existing challenges in the area of modern technological tools is related to the issue of ethics. The aim of this study is to discuss ethical concepts of executive phases of IoT in healthcare.

## 2 METHODS

The methodology applied here is that of a mixed‐method approach. A systematic review and expert consensus were used to retrieve relevant ethical issues. We adhered to the protocol to review articles based on preferred items to report in systematic reviews and meta‐analyses (PRISMA).^11^ The current study was conducted in two phases:

### 2.1 Phase 1: Identification of the ethical issues in IoT technology using the systematic review

In the first phase of this study, a systematic review was conducted in relevant databases, including PubMed, Scopus, and Web of Science, to identify appropriate ethical issues of IoT technology. Keywords that were used to search for sources of information include words related to the concepts of “internet of things” and “Ethics”. The search string is defined as follows: ((“internet of things” OR “IoT”) AND (“Ethic*”)). Articles that were published between 2013 and 2022 were selected. Our inclusion criteria were: full‐text papers with the relevant keywords in the title or abstracts, studies that were published from 2013 to February 2022, and studies published in the English language. In addition, review and systematic review articles were included in the search result, and articles that did not report any ethical issues were excluded. In the first step, the abstract and title of articles were studied according to the inclusion/exclusion criteria. Screening of titles and abstracts was conducted independently by two researchers. The disagreement between researchers was resolved by consensus. In the next step, The full texts of articles, which seemed relevant to the objectives, were reviewed by the same two researchers. Any disagreement was resolved by consensus. Finally, ethical issues were extracted from the selected articles.

### 2.2 Phase 2: Classification of the ethical issues using the focus group discussion

During the first phase of the research, the identified ethical issues employed various classifications of the data elements. Therefore, a focus group discussion was used to classify the extracted ethical issues based on executive phases of IoT in healthcare by contributing five experts affiliated with SUMS (three medical informatics and two computer engineering). They were selected due to their familiarity with IoT technology and experience in the field of medical sciences. To adhere to ethical considerations, the experts participated in the study with full knowledge of the objective of the research and could withdraw from the study at any time. This focus group was held in the department of health information management at Shiraz University of medical science. This session lasted 2 h. All extracted ethical issues were discussed with all experts’ opinions taken into account.

### 2.3 IoT system executive phases

2.3.1

IoT has five phases based on online and offline requests. These phases include collecting data to delivering data.

1. Data collection: The first step is gathering, collecting, or receiving data from devices and objects. Different data collectors based on the characteristics of objects are used. The object might be a fixed object like body sensors or radio‐frequency identification (RFID) tags or a dynamic and moving device like sensors and chips.
2. Data storage: Data collected from the previous phase should be stored. If the object has an internal memory, the data can be stored. Typically, IoT components are installed with low memories and low processing powers. Clouds will take responsibility for storing data when devices’ internal memory is unavailable.
3. Data process: IoT analyzes the data stored in the data center of the clouds and provides smart services for work and life in real‐time. In addition, IoT not only analyzes and responds to queries but also controls objects. IoT provides smart processing and controls the services of all objects identically.
4. Data transmission: Data transmission occurs in all stages: from sensors, RFID tags, or chips to data centers, from data centers to process units, processors to controllers, devices, and end‐users.
5. Data delivery: Delivering the processed data to objects at the moment without any errors or changes is an essential and sensitive task that should always be done.^12^, ^13^

## 3 RESULTS

### 3.1 Phase 1

**Figure 1:**
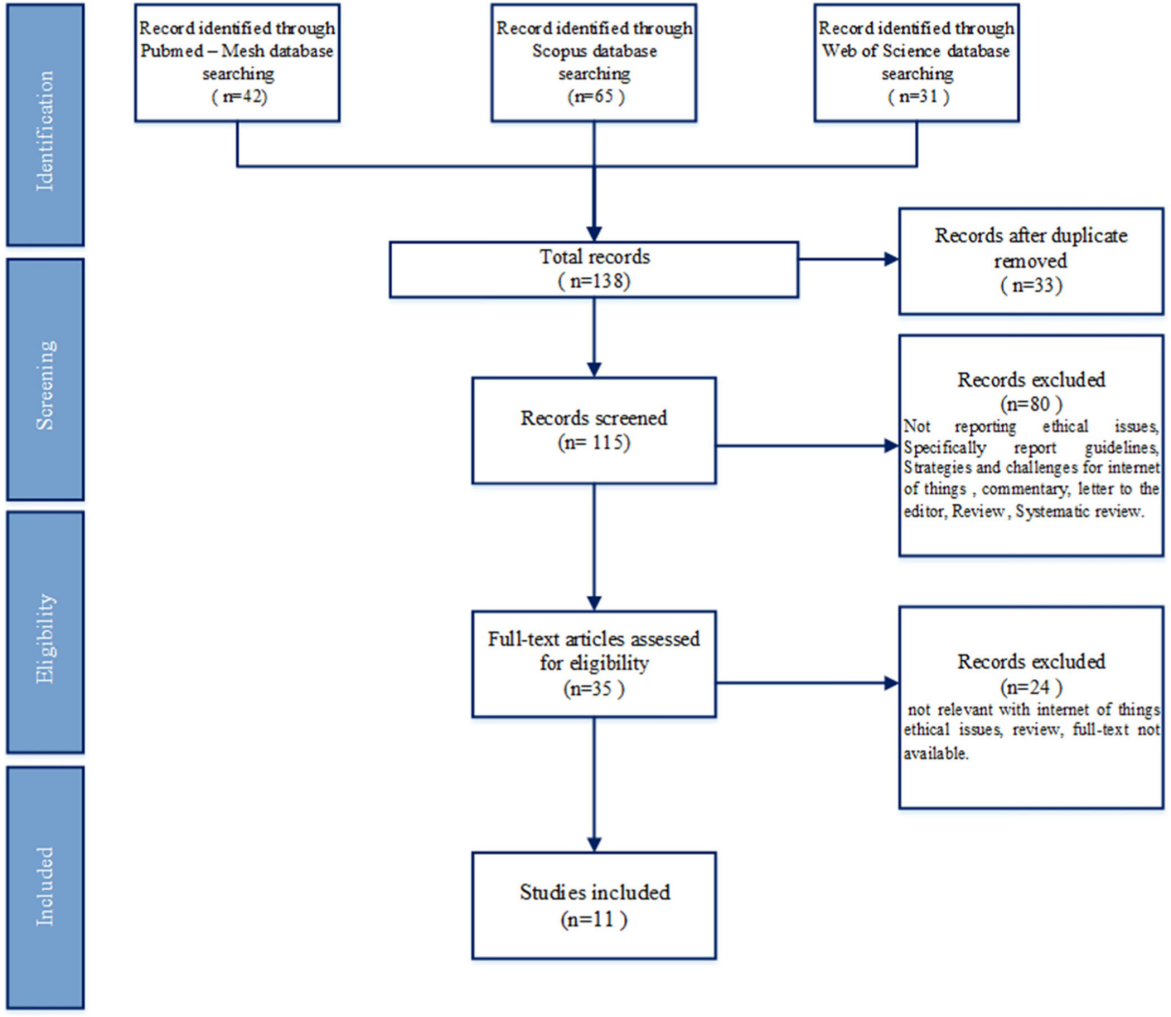
PRISMA flow diagram with the steps in the article selection process. PRISMA, preferred items to report in systematic reviews and meta‐analyses.

Based on the search strategy, a total of 138 articles were retrieved. Overall, there were 23 duplicates among the databases, which were excluded. After removing duplicates, the abstract and title of 115 articles were studied. At this stage, 80 articles were excluded, considering the irrelevance of the article title or abstract. The full texts of 35 articles seemed relevant to the objectives. In the final analysis, 11 articles were selected, and 12 ethical issues related to IoT were identified. The literature search results are shown in Figure 1, and the result of extracted ethical issues from these 12 articles are presented in Table 1.

**Table 1:** Result of extracted ethical issues from selected articles in the field of the Internet of Things

Study	Security	Confidentiality	Privacy	Anonymity	Freedom to withdraw	Informed consent	Integrity	Availability	Authorization	Access control	Censoring	Eavesdropping
Chang et al.^14^	*	*	*	*	*	*	*	*	*	*	*	*
El‐Khoury & Arikan^15^	*	*	*	*		*	*	*		*		
Baldini et al.^16^	*		*	*	*	*		*	*	*		
Karale^17^	*	*	*	*		*	*	*	*	*	*	*
Philip et al.^18^	*	*	*	*		*	*	*	*	*		
Aledhari et al.^19^	*		*			*	*	*	*	*		*
Olawole^20^	*	*	*					*		*		*
Nadian‐Ghomsheh et al.^21^	*	*	*					*		*		
Lhotska et al.^22^	*	*	*	*		*			*	*		
Popescul & Georgescu^10^	*	*	*			*	*		*	*		
Furstenau et al.^23^	*		*	*			*	*	*	*		

### 3.2 Phase 2

**Figure 2:**
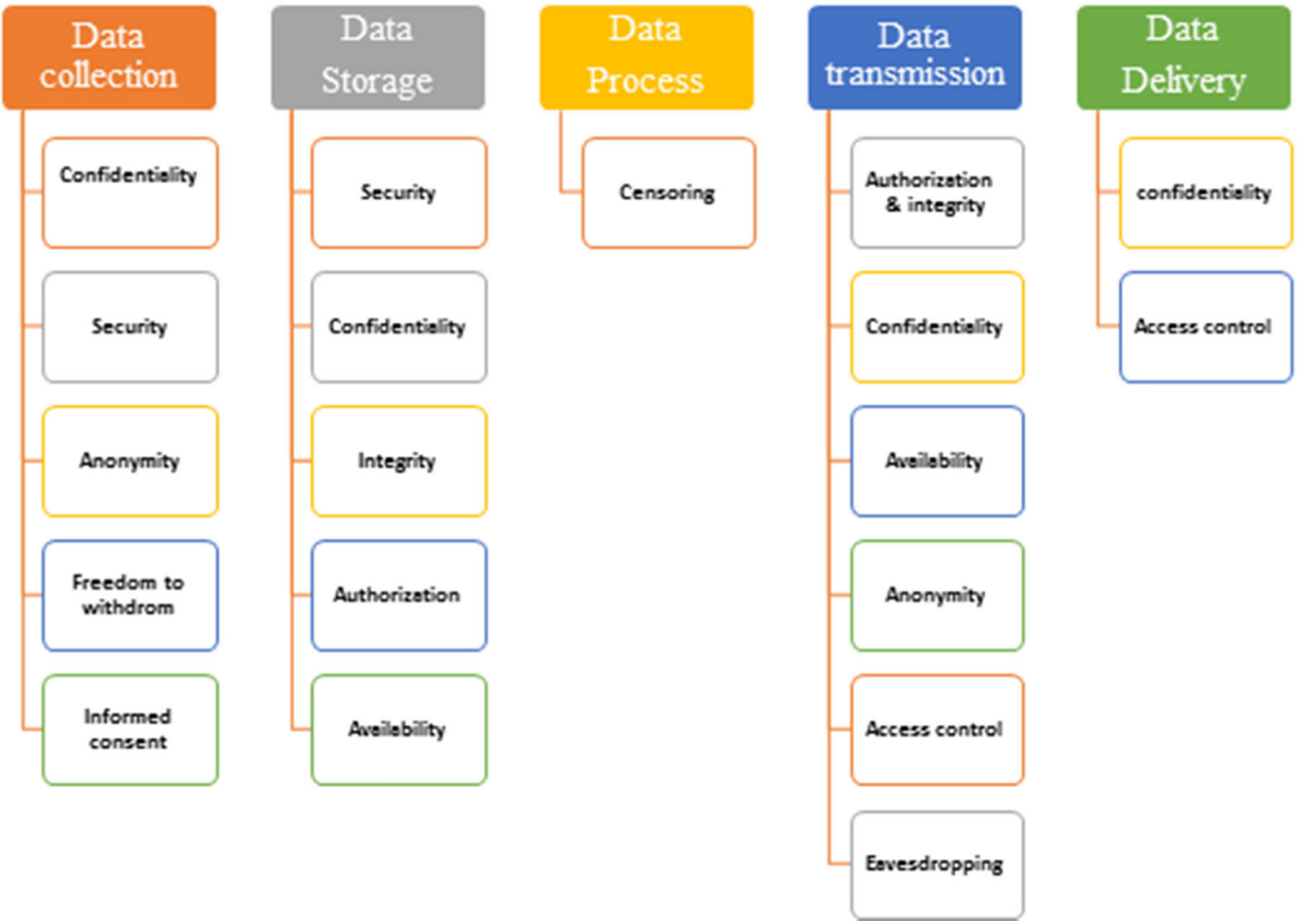
Ethical issues based on executive phases of IoT system. IoT, Internet of Things.

Based on the experts’ opinions, the ethical issues were assigned into five main categories: data collection, storage, process, data transmission, and delivery. The major categories of IoT ethical issues are summarized in Figure 2. Data collection included five elements: confidentiality, security, anonymity, freedom to withdraw, and informed consent. Data storage includes five elements: security, confidentiality, integrity, authorization, and availability. Censoring is the only item of the data process. Data transmission includes authorization, integrity, confidentiality, availability, anonymity, access control, and eavesdropping. And finally, Data delivery included two elements: confidentiality and access control.

## 4 DISCUSSION

The IoT has made great strides in recent years in all areas, especially in healthcare, which has attracted the attention of many researchers and developers worldwide. This technology, despite its advantages, has many challenges that can lead to failure or being useless. One of the most important and challenging issues in the IoT is ethical issues. Therefore, the identification of each of these issues and their solutions in accordance with the implementation phases of this technology should be considered. The categories and items obtained in the findings are discussed as follows.

### 4.1 Data collection

One of the most important issues in this area is collecting and exchanging individuals’ data with information technology. Today, many individuals are hired to collect, explore and distribute data. This large size of private information can threaten ethical issues. For example, violation of individuals’ privacy is one of these challenges. Most people's information may be used for various purposes without their awareness of individuals.^24^, ^25^

1. Confidentiality: Protecting the confidentiality of the collected data in an online study requires techniques and measures that are quite different from protecting the confidentiality of paper‐based data. Using secure sockets layers (SSL) when the data are sent to servers makes data transmission secure. Security equipment, such as encryption, can be useful measures for protecting the data on the server. Online collected data will probably be as secure as locked and protected data in research laboratories with such safety measures.^26^
2. Data security: Confidential data and information collected from participants should be safely stored, protected, and eliminated. This can be achieved through passwords, physical locks, and limiting the staff who can access the identified data.
3. Anonymity: Identifying information needs to show the consent and agreement of individuals, presenting contact information for receiving data or payment, and allocating credit for participation in research should be kept in a place separate from the data collected from that study. For instance, these data can be kept in a separate database. Therefore, in case an error occurs or data are available to individuals without permission, the data will be at least anonymous.
4. Freedom to withdraw: Freedom for participants and samples taking part in medical research should be preserved. Participants should have completely clear and obvious information on who, for what purpose, and to what extent they should have access to their data to decide to provide their data for research applications.^26^
5. Informed consent: During online data collection, researchers might never meet the participants, which suggests a challenge in informed consent. Informed consent is one of the most fundamental concepts in medical ethics and patient rights in the world, such that acquiring informed consent before any diagnostic and therapeutic activity will have positive ethical and clinical results. Informed consent is considered a major component of patient rights in healthcare centers. It is a process in which the patient or their legal representative understands and agrees to the treatment plan.^27^

### 4.2 Data storage

In the data storage process, data protection and security are considered major factors for acquiring the trust of users and successful use of cloud computing.^28^ Cloud computing has changed the environment. Right now, people are transferring their information to clouds, particularly since data have grown larger and they need more devices to be accessible. Therefore, data security and confidentiality have always been important issues in information technology.^16^ And in this situation, data security in cloud computing is becoming more and more important.^29^, ^30^

1. Data security: Data security covers four main areas: Encryption, server security, client security, and password security.
  • Encryption: One of the major components of security is encryption. SSL is an industrial encryption technology that provides online banking security and electronic commerce. SSL guarantees all communications between your computer and cloud‐based servers.
  • Server security: While SSL helps establish safe communication between your computer and a cloud, you also need to make sure that servers are safe against hackers and other threats. Although it is pretty difficult to assess the security of cloud‐based servers for web users, there are services by companies that regularly assess security on SaaS providers to ensure the security of servers.
  • Client security: Although cloud computing has the advantage of outsourcing server‐level security and backup, an overlooked part of the security equation is the security of the desktop or laptop from which you are accessing the SaaS application.
  • Password security: Finally, security also encompasses password security. The best SSL encryption and client/server security can be undone by choice of a weak password. Thus, care should be given when choosing a password.^31^, ^32^

2. Data confidentiality: Data confidentiality is very important for users to store their private or confidential data. Strategies for identification and access control are used to ensure data confidentiality. In general, all information you enter into a cloud computing application should be considered confidential and private information that cannot be used by the cloud computing provider. Furthermore, the cloud computing provider should only be permitted to view any of your private information with your explicit consent (e.g., to troubleshoot a technical issue).
3. Data integrity: Data integrity is one of the most important factors in all information systems. In general, data integrity means the protection of data against unauthorized elimination, change, or construction. Data integrity is a basis for offering cloud computing services such as SaaS, PaaS, and IaaS. In addition to storing data on a large scale, cloud computing generally provides data processing services. Such techniques can achieve data integrity as redundant array independent disks strategies and digital signatures.
4. Authorization: Authorization is used for data access control. It is a mechanism by which a system determines the level of access by valid users for working with secure sources under the control of the system.^33^, ^34^
5. Availability: Companies needing to store large‐scale data have two options: Using a local data center or storing with a cloud. If appropriately used, storing in a cloud enables such companies to use resources in various geographical regions to ensure data availability even when they face local/regional/district disasters.^35^, ^36^, ^37^

### 4.3 Data process

Cloud computing has two essential processes: (1) Data processing and computing, and (2) Data storing. In the data processing and computing process, users of cloud services do not need anything, and they can have access to their data and fulfill their computing and processing tasks only through a connection to the Internet. Clients do not even know where the data are stored during data availability and computing and what device or machine carries out the task of computing.^13^, ^32^

1. Censoring: Censoring data and findings is an important ethical issue. Censoring can occur before data collection, during data analysis, or during data reporting. Censoring can be done by audits, assessment providers, or some other stakeholders.^38^

### 4.4 Data transmission

It is necessary to protect data during data transmission because attacks on data and disclosure of data during data accessor transmission lead to the illegal use of patients’ private information.^8^

1. Authorization and integrity: This is often guaranteed by hash functions and controlling and computing each package sent between servers and censors in the network^34^, ^39^
2. Confidentiality: This often occurs through symmetric encryption on the traffic sent between servers and sensors. Ultimately, confidentiality is achieved by using automatically updating the key or the password.^40^
3. Availability: Access to services is provided for authorized and legal users, and reactions are shown to simultaneous access by a large number of users to services.
4. Anonymity: Patient privacy is vital in a healthcare system. Therefore, patient anonymity is guaranteed by the system. In this phase, data transmission should be done so that hackers will not be able to have access to the patients’ Identifiers, identify them or restore their information.
5. Access control: While access control is performed in a partial or fine‐grained manner when it comes to the data stored in servers, access control is also guaranteed in conditions where multiple individuals’ writing or editing operations occur simultaneously. In such a state of access by users to data, access is prevented before necessary access policies are checked to check if the user meets the rules.^41^
6. Eavesdropping: A hacker that eavesdrops wants to have access to patients’ private and sensitive medical data. Such hacking operations might occur during the communication between the patient and servers that provide healthcare or between servers that provide healthcare and cloud‐based servers. For this, solutions such as forced use of access control systems should be considered to prevent conspiracy by users (patients, healthcare team, and staff) aiming at unauthorized access and receipt of medical data.^41^, ^42^

### 4.5 Data delivery

With advances in the area of cloud computing, the provision of many services or computing sources for the end user is achieved through clouds. Outsourcing data is a new paradigm in which cloud computing providers can store them as a third party. This is very cost‐effective for users because they do not need to purchase expensive hardware and software for data storage. Users are also free to manage, update, upgrade, repair, and maintain software. Private data of organizations are stored in secure and reliable sites and are provided for use from anywhere based on their requests. Confidentiality, integrity, and availability are challenging issues related to storage management and data provision.^9^, ^43^

1. Access control: Users can access cloud‐based storage servers and their private information through their specific usernames and passwords. This method can prevent unauthorized users from entering servers and stealing data. Authorized users can establish communication with cloud servers to see their private information while they cannot have access to their non‐private data. This method can prevent unauthorized access by authorized users. Through a dynamic authorization mechanism, patients can sign up to receive an authorized code and access their medical data.^44^, ^45^
2. Confidentiality: This often happens by decrypting the encrypted data at data delivery.^40^ These factors were addressed in the related literature; Kelly et al.^46^ mentioned confidence, privacy and security, data storage, control, and ownership as barriers to IoT‐based health care. Also, other categorizations were seen in the literature: Malhotra et al.^47^ categorized vulnerabilities in IoT as lack of physical security, lack of network‐based security, software‐based vulnerabilities, insufficient privacy protection with default settings, and insufficient audit mechanism. In addition to general ethical issues in healthcare IoT systems, specific issues such as IoT in real‐time security issues have been addressed by Chen et al.^48^ they have mentioned injection of malicious code, network attacks, crashing the system, for example, by DoS attacks, and extracting sensitive information by side‐channel attacks. Although the authors mentioned these issues in the real‐time IoT system context, they could be valid in the general IoT context. Pal et al.^49^ categorized IoT security issues in an architectural layer into (1) device sensing layer, (2) network management layer, (3) Service composition layer, (4) application layer, and (5) user interface layer. In these layers, the common security issues include (1) authentication, authorization, and access control, (2) unauthorized access, modification of routing paths, (3) service authentication, data confidentiality, (4) unauthorized access, privacy leakage, integrity, and (5) authentication and authorization, and data confidentiality.

## 5 CONCLUSION

What is certain is the application of IoT in the future and the existence of such a network. Although the modern technology of IoT will have many achievements in healthcare systems, it will also face numerous challenges like other emerging technologies. One of these challenges is ethical issues when large amounts of patient data are collected by objects and data computing, analyzing and storing these data by cloud‐based servers and transferring these data through the network. As illustrated by the present study results, the most important ethical issues of medical IoT included security, access control, and privacy. These ethical challenges should be considered by employees and managers of organizations that use this technology. In addition to the fact that health policymakers should be aware of the moral obligation to use this technology, they should establish strict standards, regulations and policies for the use of these devices in the field of health and treatment. For example, to minimize network attacks and unauthorized access to information in the IoT, solutions such as correct configuration of application server settings, holding confidentiality and privacy training courses, determining authorized access levels and using secure information platforms are suggested.

## AUTHOR CONTRIBUTIONS

**Somayyeh Zakerabasali**: Conceptualization; data curation; formal analysis; investigation; methodology; supervision; writing – original draft; writing – review & editing. **Seyed Mohammad Ayyoubzadeh**: Data curation; investigation; writing – original draft.

## CONFLICT OF INTEREST

The authors declare no conflict of interest.

## TRANSPARENCY STATEMENT

The lead author Somayyeh Zakerabasali affirms that this manuscript is an honest, accurate, and transparent account of the study being reported; that no important aspects of the study have been omitted; and that any discrepancies from the study as planned (and, if relevant, registered) have been explained.

## Data Availability

The authors confirm that the data supporting the findings of this study are available within the article and/or its Supporting Information.
